# Recovery and evaluation of cellulose from agroindustrial residues of corn, grape, pomegranate, strawberry-tree fruit and fava

**DOI:** 10.1186/s40643-021-00377-3

**Published:** 2021-04-02

**Authors:** Mariana Vallejo, Rachel Cordeiro, Paulo A. N. Dias, Carla Moura, Marta Henriques, Inês J. Seabra, Cândida Maria Malça, Pedro Morouço

**Affiliations:** 1 College of Agriculture, Polytechnic of Coimbra, 3045-093 Coimbra, Portugal; 2 Centre for Rapid and Sustainable Product Development, Polytechnic of Leiria, 2430-028 Marinha Grande, Portugal; 3 Chemistry Department, University of Coimbra, 3004-531 Coimbra, Portugal; 4 CIEPQPF, Department of Chemical Engineering, University of Coimbra, 3030-790 Coimbra, Portugal; 5 Bioengineering Department, Lehigh University, Bethlehem, PA USA; 6 Institute of Engineering, Polytechnic of Coimbra, 3045-093 Coimbra, Portugal; 7 ESECS, Polytechnic of Leiria, 2411-901 Leiria, Portugal

**Keywords:** Agroindustrial residue valorization, Cellulose, Solvent extraction, ATR-FTIR, TGA–DSC

## Abstract

Considering the expected increasing demand for cellulose fibers in the near future and that its major source is wood pulp, alternative sources such as vegetable wastes from agricultural activities and agro-food industries are currently being sought to prevent deforestation. In the present study, cellulose was successfully isolated from six agroindustrial residues: corncob, corn husk, grape stalk, pomegranate peel, marc of strawberry-tree fruit and fava pod. Cellulose fibers were characterized by Fourier-transform infrared spectroscopy, thermogravimetric analysis, stereomicroscopy and scanning electron microscopy (SEM). Despite the evident morphological differences among the extracted celluloses, results revealed similar compositional and thermal properties with the wood-derived commercial microcrystalline cellulose used as a control. Trace amounts of lignin or hemicellulose were detected in all cellulose samples, with the exception of corncob cellulose, that exhibited the greatest extraction yield (26%) and morphological similarities to wood-derived microcrystalline cellulose, visible through SEM. Furthermore, corncob cellulose was found to have thermal properties (T_Onset_ of 307.17 °C, TD of 330.31 °C, and Δ*H* of 306.04 kJ/kg) suitable for biomedical applications.

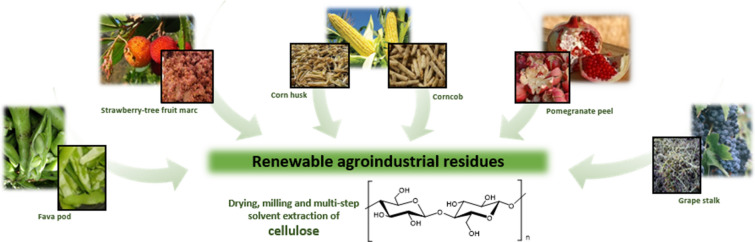

## Introduction

In the upcoming years, the demand for cellulose fibers is expected to exceed the available supply, considering the continual research that has led to the development of novel cellulose-based products, particularly in the food and biomedical fields (Hindi 2017; Abdul Khalil et al. 2020). Wood pulp remains the most popular source of cellulose due to its abundancy and cost-effective extraction which enable a large-scale production. However, the processes involved entail several environmental impacts, namely forest devastation and consequent contribution to global warming, which have driven researchers to seek environmentally friendly and biocompatible materials as sustainable alternatives to wood-derived cellulose (Pennells et al. 2020).

Currently, tissue engineering is one of the research fields that is exploring cellulose as a raw material to be applied in the formulation of biomaterials to replace or contribute to the regeneration of biological tissues such as skin, bone and cartilage (Hickey and Pelling 2019). Cellulose has several features that provide significant improvements for biocomposites, such as nontoxicity, biocompatibility, biodegradability, low cost and high mechanical modulus (Carlström et al. 2020; Naseri et al. 2016; Sultan and Mathew 2019). Since its first appearance in scaffolds, it has made significant contributes to enhance tissue engineering constructs, even when 3D printed (Müller et al. 2006). In an effort to seek alternative sources for wood-derived cellulose, cellulose derived from bacteria and chemical synthesis has recently been the focus of attention of the high-tech industry. These are very attractive pathways when the purpose is to achieve highly specific and crystalline cellulose structures (Sharma et al. 2019). Still, these are very expensive and low-yield techniques, thus representing a major shortcoming when large-scale production is envisaged.

Non-wood cellulosic biomass sources, such as agricultural and industrial wastes, offer an attractive alternative to wood pulp. Besides being inexpensive, readily available and renewable, these non-wood sources are also particularly promising, because they usually have lower lignin and higher hemicellulose contents when compared to forestry materials, leading to lower energy and chemical consumption in delignification processes and fiber purification. Considering the low economic value of the existing applications and the high value of potential cellulose products, there is a significant opportunity to convert these biomass resources into valuable raw materials. As the pulp industry follows the current trend of pursuing sustainable practices, it will further boost research on sustainable biomass procurement, further promoting circular economy practices to achieve environmentally sustainable economic growth (Nechyporchuk et al. 2016; Gontard et al. 2018).

Fava bean (*Vicia faba*) is a grain legume known for its nutritional value in terms of protein and energy (Millar et al. 2019), being widely cultivated in the Mediterranean region, India, Pakistan and China. In Portugal, the 2018 annual production of fava bean was around 4.3 thousand tons (INE 2019). In the human diet, it is mostly the seed grain that is consumed, which is separated from the pod by a decortication process. The separated pod represents around 70% of the whole fava bean weight (Basterrechea and Hicks 1991), a relevant by-product that is discarded as waste or used for animal feed (Crépon et al. 2010). However, considering its cellulose content (Gómez et al. 2017), fava bean pods might be considered as a possible source of these fibers.

Strawberry tree (*Arbutus unedo* L.), an indigenous species from the Mediterranean region, is present in Portugal with special prevalence in the Algarve region. Several parts of this plant have traditionally been used in medicine and the fruit is used to produce alcoholic beverages and food products. The recent increased interest in improving the quality of the fruit and the range of applications is contributing to rise the availability of by-products from the industries involved (Anjos et al. 2020; Šic Žlabur et al. 2020). In particular, the fruit marc that results from fermentation and/or pulp extraction may be tested as a source of cellulose fibers.

Corn (*Zea mays* L.) is the most produced grain crop worldwide, being a staple food for many populations and one of the main components of livestock feed. Data compiled by Statista (2020) indicate that the European Union produced 61 million metric tons of corn in the 2018/19 season, while in Portugal, production reached 714 thousand tons in 2018 (INE 2019). Considering that corncob and corn husk are 15% and 14% of the whole corn, respectively (Reddy and Yang 2015), large amounts of these agricultural residues are produced annually, which are typically used for the production of animal feed, fertilizers or simply discarded. Yet, both residues may be considered as reliable sources of cellulose fibers (de Andrade et al. 2019).

The pomegranate (*Punica granatum* L.) tree has been widely cultivated in warm regions such as India, Spain, Israel and the United States (Mujtaba et al. 2016). In Portugal, it is mostly cultivated in the south, with special prevalence in the Algarve region, where the production of pomegranate fruit reached 400 tons in 2015 (Agrotec 2015). The peel can reach up to 30–40% of the total weight of this fruit, being an abundant residue of the pomegranate juice processing industry. Due to its high content of antioxidants, this by-product is considered valuable in the food and pharmaceutical industries, where it can be applied as food additive or as a source of dietary fiber (Singh et al. 2018). However, and considering its relatively high cellulose content (17–22%, in weight) (Mujtaba et al. 2016), it may also be used as a possible source of cellulose.

Lastly, grape vine (*Vitis vinifera* L.) is an ancient and wide-spread culture in Portugal. Winemaking is a particularly relevant sector of the Portuguese economy, with a wine production of around 5.9 million hectoliters in 2018 (INE 2019). Prior to grapes pressing, during the sorting process, stalks are separated from the grapes and discarded. Considering that the production of 100 L of wine generates approximately 4 kg of stalks, approximately 23.5 thousand tons of grape stalks are generated in Portugal per year (Ferreira et al. 2018). Currently, this residue does not have any practical application except for being used as a fertilizer. However, viable alternatives have been studied, such as the extraction of cellulose and hemicellulose (Spigno et al. 2008).

The focus of the present study was to evaluate the cellulose isolation suitability from the six aforementioned and renewable agroindustrial residues available in Portugal, namely fava pod, marc of strawberry-tree fruit, corncob and corn husk, pomegranate peel, and grape stalk, following a multi-step solvent extraction procedure. The isolated cellulose samples were characterized and compared to wood-derived microcrystalline cellulose, considering their further application in the biomedical field as a reinforcing material of composite scaffolds.

## Materials and methods

### Materials

Corncob and corn husk were kindly provided by a local farmer, and grape stalks were provided by a local winery, both located in the center region of Portugal (Coimbra, Portugal). Strawberry-tree fruits were picked in the center region of Portugal, and provided by Lenda da Beira Lda (Pampilhosa da Serra, Portugal). Pomegranates were provided by POM Portugal Lda (Beja, Portugal), and deseeded fava bean pods were provided by Vitacress Portugal SA (Odemira, Portugal).

Analytical grade chemicals and solvents employed for the multi-step extraction procedure and analysis of the isolated cellulose samples were ethanol (100%) from Chemlab, sodium hydroxide (99.9%), sodium bisulfate (95%) and sodium chlorite (80%) from Honeywell Fluka, nitric acid (75%), acetic acid (99–100%) and toluene (99%) from BioChem, and microcrystalline cellulose (Avicel PH102).

### Raw material preparation

Ripened strawberry-tree fruits were subjected to pulp extraction. Initially, fruits were crushed using a blender (Braun MQ5137 BK, Germany) and then exposed to enzymatic hydrolysis with pectinase (Pectinex, Novozymes), followed by manual pressing/filtration using a 0.1–0.2 mm polyamide cloth to efficiently separate fruit pulp from pomace, mainly composed by fruit seeds and sclereids. Strawberry-tree fruit marc was stored at − 20 °C until further processing.

Ripened pomegranates were rinsed with tap water and sorted to remove rotten fruits. Fruits were cut into pieces, and the arils separated from the peels using a grape stem removing machine (COSVAL, Mizar 60, Portugal). Pomegranate peels were stored at − 20 °C until further processing.

Raw materials (fava pods, thawed strawberry-tree fruit marc, corncob and corn husk, pomegranate peels and grape stalks) were dried in a pilot-scale tray dryer (STI-SDP, Portugal) at 50 °C, under tangential hot air convection at 1.5 m/s and 30% relative humidity, for approximately 20 h. The dried raw materials were then ground using a cutting mill SM 100 (Retsch, Germany) equipped with a sieve with an aperture size of either 0.5 mm (for corn husk and strawberry-tree fruit marc) or 2.0 mm (for the remaining raw materials).

Moisture content of the raw materials was determined gravimetrically through oven-drying for approximately 12 h at 105 °C, with triplicate assays.

### Multi-step cellulose isolation process

**Fig. 1:**
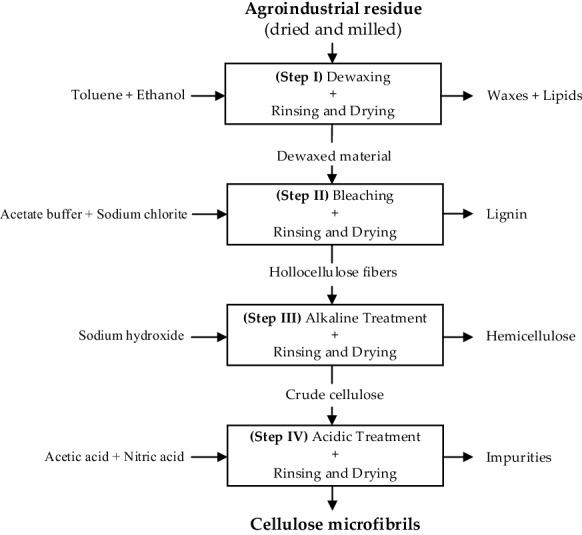
Simplified flow diagram of the cellulose isolation process applied to the agroindustrial residues

The fractionation process used for cellulose isolation from the six agroindustrial residues was optimized based on several sources (Sun et al. 2004; Morán et al. 2008; Maheswari et al. 2012; Madureira et al. 2018). Figure 1 shows the schematic representation of the four sequential extraction steps applied.

At the first fractionation step, the raw materials were dewaxed for 6 h in a Soxhlet apparatus, using a 2:1 (v/v) toluene-ethanol solvent mixture and a solid-to-solvent ratio of 1:20 (w/v). The dewaxed raw materials were then rinsed with ethanol for 30 min under vacuum filtration, and dried in an oven (Memmert, Germany) at 105 °C until constant weight. Next, the dried dewaxed raw materials went through a bleaching step for delignification purposes using a 1:1 (v/v) mixture of acetate buffer and sodium chlorite aqueous solution (17 g/L) for 4 h, at 100 °C, using a solid-to-solvent ratio of 1:40 (w/v). The delignified celluloses were then consecutively rinsed with a solution of sodium hydrogen sulfate (20 g/L), deionized water and ethanol under vacuum filtration and subsequently dried at 105 °C. The resulting crude holocelluloses were then subjected to alkaline treatment with a sodium hydroxide solution (175 g/L) in an orbital shaker (Unimaz 1010, Heidolph, Germany) for 45 min, at room temperature and 60 rpm, and using a solid-to-solvent ratio of 1:50 (w/v). The crude cellulose fibers were subsequently rinsed with 10% acetic acid, followed by deionized water. After being dried at 105 °C, the crude cellulose fibers were finally submitted to an acidic treatment to remove impurities. A 10:1 (v/v) mixture of acetic acid (80%, v/v) and nitric acid (70%, w/v) was used at 120 °C (in an oil bath) for 15 min with a solid-to-solvent ratio of 1:40 (w/v). After rinsing with ethanol, distilled water and ethanol again (under vacuum filtration) to remove the remaining traces of acid, the purified cellulose fibers were dried at 105 °C. This multi-step extraction process was repeated at least three times for each agroindustrial residue considered in this study.

The percentage of material removed at a given step *i* relatively to the initial raw material mass, Rm_*i*_, was calculated by Eq. 1, and the cellulose extraction yield, Yield_cellulose_, was determined by Eq. 2.

1 $${\text{Rm}}_{i} \left( \% \right) = \frac{{m_{i} - m_{i - 1} }}{{m_{0} }} \times 100$$

2 $${\text{Yield}}_{{{\text{cellulose}}}} \left( \% \right) = \frac{{m_{4} }}{{m_{0} }} \times 100,$$

where *m*_*0*_ is the dry mass of the pre-treated agroindustrial residue, *m*_*i−1*_ is the dry mass of the material obtained at the end of step *i−1*, and *m*_*i*_ is the dry mass of the material obtained at the end of step *i* (*i* = 1, …, 4).

### Attenuated total reflectance Fourier-transform infrared (ATR-FTIR) spectroscopy analysis

The ATR–FTIR spectra of the samples obtained at the end of the multi-step extraction process were recorded to confirm the presence of cellulose and its purity. Samples were powdered in a mortar and mixed with a small volume of sodium hydroxide solution (0.1 M) to get a paste to be processed into a pellet suitable for analysis (approximately 1 mm thickness). A Bruker Alpha-P FTIR spectrometer was used in the transmittance mode, with ATR platinum–diamond coupling. The samples were analyzed at ambient temperature, 4 cm^−1^ spectral resolution, at 64 scans per sample, and in the range 4000–400 cm^−1^. Pure microcrystalline cellulose (MC) was used as control.

### Thermal gravimetric analysis (TGA) and differential scanning calorimetry (DSC)

Thermal analysis of the cellulose samples was performed using TGA and DSC on a Simultaneous Thermal Analyzer, STA 6000 system (Perkin Elmer, USA). The dried samples (approximately 9.6 mg) were weighed into alumina pans and heated from 30 to 600 °C at 10 °C/min, under a nitrogen flow rate of 20 mL/min. The mass loss of the samples and enthalpy curves, obtained as a function of temperature, were recorded and analyzed using the software PyrisTM, and compared to pure MC. This analysis was performed in triplicate.

### Morphological analysis of cellulose

The macroscopic analysis of the different isolated celluloses was assessed by digital imaging. Images of the samples were collected using a Cannon EOS 50DO supported 30 cm high in a HAISER RTX. The lightning system was placed at 17/18 cm and 45° from the center. The cellulose samples, placed in the center, were photographed under the following conditions: ISO 400, amplitude F/6.3 and exposition 1–80, with no flash.

Particle morphology was accessed by optical microscopy using a stereomicroscope (Zeiss Stemi 2000-C, Germany) to obtain 20× amplified images.

The structure and surface morphology of the most promising cellulose sample (higher extraction yield and purity) were accessed by scanning electron microscopy (SEM). Cellulose micrographs were collected at an acceleration voltage of 15,000 kV using a FEG-ESEM / FEI Quanta 400 FEG-ESEM (Waltham, EUA). Pure MC was used as control.

### Statistical analysis

Cellulose extraction yields from the six different agroindustrial residues were compared using one-way analysis of variance (ANOVA) with Tukey’s honestly significant difference (HSD) post hoc test. Two-way ANOVA, with Fisher’s test, was applied in the analysis of the mass removed in the different steps of the extraction process and in the TGA/DSC analysis. Statistically significant differences were considered at a p value lower than 0.05. The statistical analysis software was GraphPad Prism 6 (GraphPad Software, Inc.).

## Results and discussion

The moisture content of the agroindustrial residues at the end of the drying and milling processes that preceded the multi-step extraction procedure is reported in Table 1. The relative high moisture content of pomegranate peel (13.3%) may be related to its high content of soluble carbohydrates (sugars) (Gullón et al. 2020), that are usually associated with higher hygroscopicity and consequent difficulties during drying (Roos 1995).

**Table 1:** Raw materials’ moisture content

Raw material	Moisture (%)
Fava pod	11.3 ± 0.97
Strawberry-tree fruit marc	10.1 ± 0.03
Corncob	6.64 ± 0.10
Corn husk	7.48 ± 0.11
Pomegranate peel	13.3 ± 0.33
Grape stalk	8.04 ± 0.06

### Multi-step cellulose isolation process

**Fig. 2:**
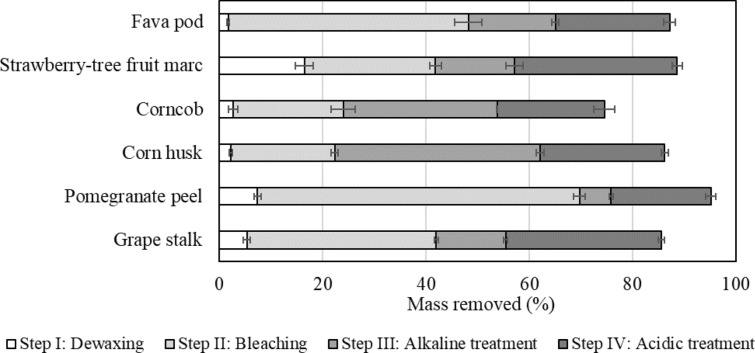
Mass removed after each extraction step (Rm_*i*,0_, %), calculated by Eq. 1

To evaluate and compare the fractionation process for each agroindustrial residue, the percentage of mass removed at each extraction step was quantified and is presented in Fig. 2. Although the relative amount of a given group of chemical compounds can be roughly observed by analyzing the mass removed at a particular extraction step, the analysis must take into consideration that each fraction may contain other chemical compounds besides the target ones, and that a certain chemical group may be removed at more than one extraction step. Nevertheless, based on previous studies (Sun et al. 2004; Morán et al. 2008; Maheswari et al. 2012; Madureira et al. 2018), it is expected that the fractions obtained at the end of steps I, II and III of the extraction procedure are rich in waxes/lipids, lignin and hemicellulose, respectively, leaving the purified cellulose isolated from each agroindustrial residue, after a final acidic treatment (Fig. 1).

During dewaxing (step I), the mass loss observed for fava pod was less than 2%, a value that is of the same order of magnitude of the lipid content found in literature for this vegetable material (Mejri et al. 2018). Low mass losses (~ 2.5%) were recorded for corn husk and corncob, which are in line with previous studies (Yeasmin and Mondal 2015; Pointner et al. 2014). Grape stalk and pomegranate peel presented wax/lipid contents of 5.4 ± 0.7% and 7.4 ± 0.7%, respectively, which are slightly smaller than the ones reported previously, 8.16% for grape stalk (Sousa et al. 2014) and 9.4% for pomegranate peel (Ullah et al. 2012). Several factors may have contributed to these differences, including some related with the plant itself (such as plant species/variety, plant maturity, geographical origin, harvesting season), and with the different extraction conditions applied, in particular the solvents used (hexane and petroleum ether versus toluene and ethanol for the present study). The lipid content of the strawberry-tree fruit marc (16.5 ± 1.70%) was considerably higher than the one reported by Ruiz-Rodríguez et al. (2011) for the whole fruit (less than 0.8%). This is an expected result because the strawberry-tree fruit marc, obtained as a result of a pulp extraction process, is mainly composed by the seeds and outer tissues of the fruit which are lipid storage structures that serve as a source of energy during embryonic development, and play a central role in sensing and interacting with the surrounding environment (Lara et al. 2015), respectively.

At the bleaching step (step II), whose purpose was the delignification of the dewaxed raw materials, the two highest losses of material during the entire extraction process occurred (Fig. 2). Pomegranate peel was the residue that presented the highest mass loss (62.4 ± 1.1%), followed by fava pod (46.5 ± 2.6%), grape stalk (36.7 ± 0.45%), strawberry-tree fruit marc (25.3 ± 1.1%), corncob (21.2 ± 2.3%) and corn husk (20.2 ± 0.67%). Lignin contents for these plant materials presented in the literature are as follows: 21–42% for pomegranate peel (Hasnaoui et al. 2014), 8–12% for fava pod, depending on the geographical origin (Malushi et al. 2017), 32.35% for grape stalk (Amendola et al. 2012), 11.9% for corncob (Pointner et al. 2014), and 2–16% for corn husk (Mendes et al. 2015). Despite the several factors that are at play and may influence the final result, both related with the plant itself and with the extraction procedure followed, and that were already mentioned when the lipid/wax contents were discussed, mass losses at this step were considerably higher than those reported in the literature for lignin contents, which seems to indicate that other chemical compounds besides lignin have been removed.

The alkaline treatment (step III) was applied with the intent of removing hemicellulose leaving crude cellulose isolated for each agroindustrial residue. Corn husk and corncob were the most susceptible residues to alkaline treatment presenting the highest mass losses, 39.7 ± 0.70% and 29.8 ± 0.13%, respectively (Fig. 2). Those values were in accordance with the hemicellulose contents reported for corn husk, reaching 37.5% (Mendes et al. 2015), and for corncob, reaching 31–33% (Neto et al. 2013). There were no statistical differences (*p* > 0.05) between fava pod (17 ± 0.7%) and strawberry-tree fruit marc contents (15.3 ± 1.7%), but with grape stalk (13.4 ± 0.4%) the differences were significant (*p* < 0.05). According to Mateos-Aparicio et al. (2010), the hemicellulose content of fava beans is around 9%, a much lower value than the one obtained in this study. However, the higher hemicellulose content of fava bean pod may be explained by the fact that the pod is the most fibrous part of the whole fava bean, having the purpose of protecting the inside seeds from physical injuries. To the best of our knowledge, no reference is found in the literature concerning the hemicellulose content of strawberry-tree fruit marc. Grape stalk presented a very similar hemicellulose content compared to previous studies (Spigno et al. 2008) that reported 14.93%. On the other hand, for pomegranate peel, a content of hemicellulose combined with pectin of approximately 8.1% was reported (Pereira et al. 2016), which is comparable to the value obtained in this work (6.1 ± 0.4%), making this residue the one with the lowest content of hemicellulose among all the agroindustrial residues considered.

In step IV, an acidic treatment was applied to the crude cellulose resulting from the alkaline treatment, thus allowing obtaining purified cellulose. From Fig. 2, it is observed that the mass loss at this stage was, by decreasing order: 31.5 ± 1.0% for strawberry-tree fruit marc, 30.1 ± 0.6% for grape stalk, 24.2 ± 0.7% for corn husk, 22.1 ± 1.1% for fava pod, 20.8 ± 2.0% for corncob, and finally 19.3 ± 0.9% for pomegranate peel.

**Fig. 3:**
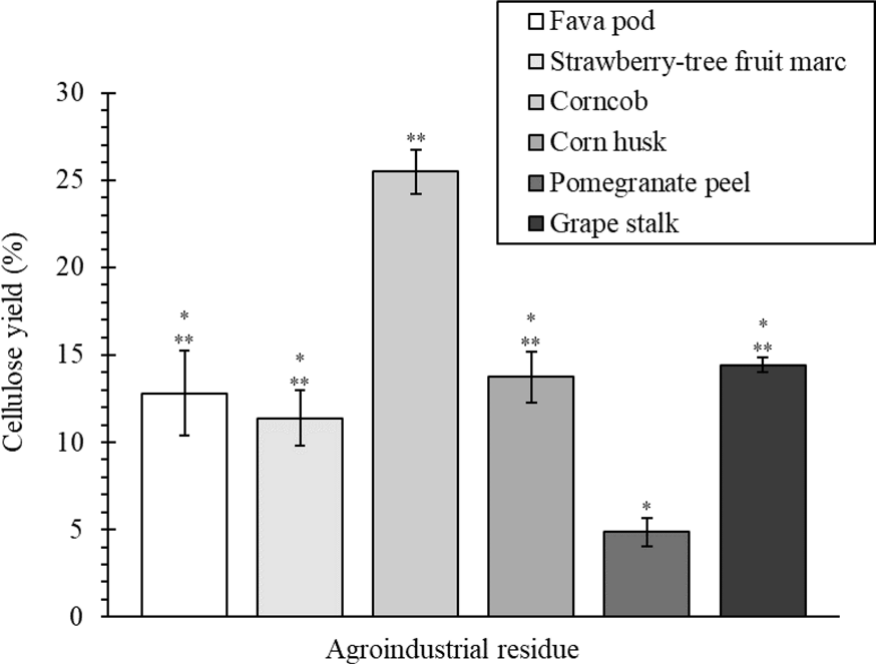
Overall extraction yield of cellulose from the six agroindustrial residues, calculated by Eq. 2

The cellulose yield, obtained at the end of the multi-step extraction process for each agroindustrial residue, is shown in Fig. 3. The influence of the rinsing and drying phases that were performed in between the extraction steps (Fig. 1) on cellulose yield was considered, and it was found that the mass losses during those operations varied between 0.2 and 0.4 g (dry basis) which may have possibly decreased cellulose yields from 2 to 4%.

Corncob presented the highest cellulose yield, reaching 26.1 ± 1.2%. The cellulose content in corncob was previously reported to range from 31 to 39% (Pointner et al. 2014), which suggests relevant material losses during the extraction process, beyond the already mentioned maximum of 4% losses during the rinsing/drying steps. It is also important to notice that the cellulose isolation process applied in this work had the intention to obtain high-purity cellulose for biomedical applications. That requested severe treatments such as the bleaching step (where material losses were pronounced) and the acidic treatment for cellulose purification, identified as responsible for the cleavage of the amorphous regions of cellulose (Rojas et al. 2015). This could explain the lower cellulose content found in this work for some raw materials in comparison to the ones reported in literature. Fava pod, strawberry-tree fruit marc, corn husk and grape stalk presented similar (*p* > 0.05) cellulose yields, between 11.4 and 14.4%. According to previous studies, the cellulose content of fava pod is around 8.33% (Mateos-Aparicio et al. 2010), 7% for strawberry-tree fruit marc (Özcan and Haciseferoğullari 2007), from 30 to 45% for corn husk (de Carvalho Mendes et al. 2015; Mendes et al. 2015; Yeasmin and Mondal 2015) and between 12 and 38% for grape stalk (Spigno et al. 2008; Amendola et al. 2012), depending on the extraction process used and also on the plant-specific characteristics. While the results obtained for fava pod, strawberry-tree fruit marc and grape stalk are in line with the reported ones, in the case of corn husk, the value was significantly lower, which probably indicates the occurrence of relevant cellulose losses during the fractionation process. Finally, the lowest cellulose yield was recorded for pomegranate peel (4.8 ± 0.8%), which is considerably smaller than the value previously reported of 17% (Malviya et al. 2014; Mujtaba et al. 2016). In this case, the great mass losses observed during bleaching may explain the reduced amount of the recovered cellulose.

### ATR-FTIR spectroscopy analysis

**Fig. 4:**
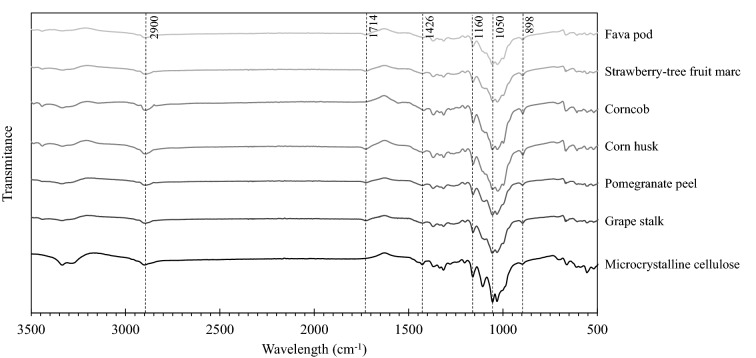
FTIR spectra of the six extracted celluloses and MC in the region 3500–500 cm^−1^

Figure 4 shows the FTIR spectra of the samples obtained in this study and of the wood-derived commercial MC. All spectra presented roughly the same peaks, indicating the presence of the same functional groups and confirming that the multi-step extraction process was successful in isolating purified cellulose from the six agroindustrial residues.

All samples presented a peak at around 2900 cm^−1^ that is characteristic of cellulose and corresponds to C–H stretching vibration. Additional bands associated to cellulose and detected in all samples were obtained at 1160 cm^−1^, assigned to asymmetric C–O–C bridge stretching, and at around 1050 and 898 cm^−1^, corresponding to C–O stretching and C–H vibrations, respectively (Morán et al. 2008; Madureira et al. 2018). The band assigned to symmetric CH_2_ bending vibration at 1426 cm^−1^, known as the crystallinity band and whose intensity is directly related with a higher degree of cellulose crystallinity (Kalita et al. 2013), was more pronounced in the MC and corncob samples.

Additionally, in all samples with the exception of the MC and corncob cellulose, a small peak at around 1714 cm^−1^ was detected that can be assigned to aromatic compounds in lignin, or to ketone and carbonyl groups in hemicellulose (Colom et al. 2003; Morán et al. 2008). Other peaks specific of lignin and hemicellulose were not detected, which seems to indicate that only trace amounts of these compounds may eventually be present in those cellulose samples.

### TGA and DSC analyses of cellulose

The thermal stability of cellulose samples is an important parameter to be considered to evaluate their applicability in new formulations that require high-temperature processing, such as new composite materials intended for biomedical applications. In general, the thermal degradation of lignocellulosic materials begins with an early decomposition of hemicellulose, followed by an early stage of pyrolysis of lignin, depolymerization, active flaming combustion, and char oxidation (Rojas et al. 2015). According to the literature, the thermal decomposition of hemicellulose and some portions of lignin occurs usually between 250 and 300 °C, while cellulose, a polymer that does not present a melting point, suffers degradation at temperatures in the range 300–350 °C (Dhyania et al. 2020).

**Fig. 5:**
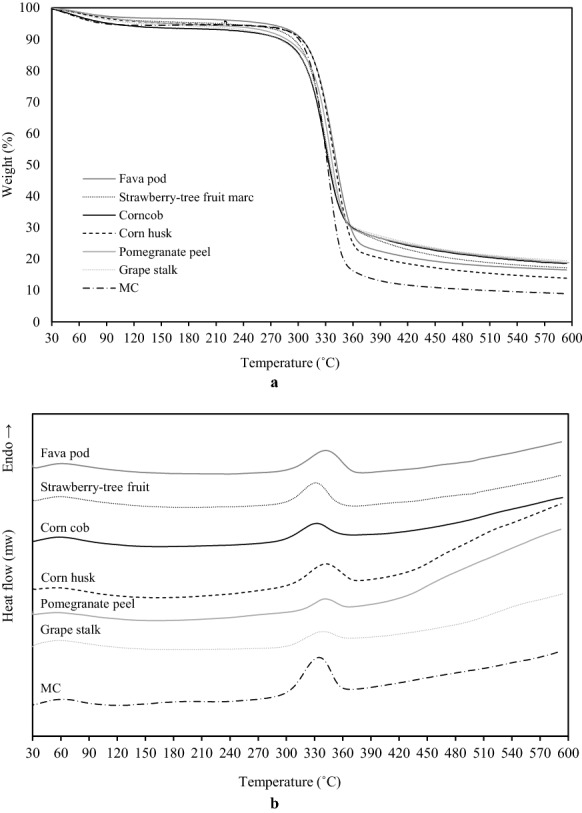
TGA (**a**) and DSC (**b**) curves of the six extracted celluloses and microcrystalline cellulose (MC)

Figure 5 shows the TGA and DSC curves for all cellulose samples, including MC, and Table 2 reports the data obtained from these curves. The TGA curves of the MC and of the six cellulose samples obtained in this study were similar. A small weight loss was found in the range of 30–150 °C, due to the evaporation of low molecular weight compounds and water, and ranged from 4.1 to 5.3%, being of the same order of magnitude of the one observed for the commercial MC (5.61 ± 0.11%) (Table 2). No noticeable thermal events were observed after water evaporation and up to 306.4–316.7 °C, depending on the agroindustrial residue, confirming the effectiveness of the extraction procedure in removing hemicellulose and lignin from the raw materials. However, the variation of the onset temperatures between 306.4 and 316.7 °C (Table 2) might be associated with residual amounts of hemicellulose and lignin, confirming the hypothesis presented in the FTIR analysis.

**Table 2:** Water loss, onset temperature (*T*_Onset_), degradation temperature (*T*_D_), decomposition enthalpy (Δ*H*_Decomp_), and total mass loss (TML) obtained from TGA and DSC analyses of the six cellulose samples

Raw material	Water loss (%)	*T*_Onset_ (°C)	*T*_D_ (°C)	Δ*H*_Decomp_ (kJ/kg)	TML (%)
Fava pod	4.95 ± 0.11	314.02 ± 0.89	339.65 ± 0.73	306.04 ± 8.38	86.71 ± 0.63
Strawberry-tree fruit marc	4.69 ± 0.17	309.11 ± 0.34	329.62 ± 0.09	254.60 ± 9.13	82.73 ± 0.25
Corncob	5.19 ± 0.29	307.17 ± 1.60	330.31 ± 0.19	166.55 ± 4.36	82.14 ± 1.47
Corn husk	4.98 ± 0.19	316.69 ± 1.34	339.40 ± 0.43	259.66 ± 8.88	84.83 ± 1.42
Pomegranate peel	4.11 ± 0.24*	311.24 ± 0.62	333.65 ± 0.32	89.75 ± 2.45	80.05 ± 0.44
Grape stalk	5.29 ± 0.37	306.38 ± 0.53	330.79 ± 0.74	125.47 ± 5.94	81.97 ± 1.66
MC	5.61 ± 0.11	312.24 ± 0.58	332.50 ± 0.46	413.74 ± 9.84*	91.15 ± 0.47***

Mean value ± standard deviation. *n* = 3. *p* < 0.05 (*) and *p* < 0.001 (***)

*MC* microcrystalline cellulose

The degradation temperatures were within the range of 330–340 °C as expected for cellulose-rich materials (Morán et al. 2008), and in line with the degradation temperature found for MC (332 ± 0.46 °C). The materials that presented higher thermal stability were fava pod cellulose and corn husk cellulose, with degradation temperatures reaching 340 °C. This is another indication that the multi-step extraction process applied in this study was efficient in isolating purified cellulose from the six agroindustrial residues, considering that cellulosic materials containing significant percentages of lignin and hemicellulose have lower degradation temperatures (Jakab et al. 2018) due to the stronger chemical bonds of cellulose that require more energy to break. Although the MC degradation temperature was within the range of the studied cellulose samples, the energy required to decompose it was much greater (413.74 ± 9.84 kJ/kg) (Table 2), which might be associated with its higher crystallinity degree and consequent greater thermal stability (Poletto et al. 2014). Among the cellulose samples obtained in this study, fava pod cellulose required the highest amount of energy to decompose (306.04 ± 8.38 kJ/kg), followed by corn husk, strawberry-tree fruit marc, corncob and grape stalk celluloses. The lowest value was found for pomegranate peel cellulose (89.75 ± 2.45 kJ/kg).

The total mass loss (TML) at the end of the thermal analysis is also reported in Table 2. Among the agroindustrial celluloses, the highest TML was obtained for fava pod (86.71 ± 0.63%) and the lowest for pomegranate peel (80.05 ± 0.44%), being in all cases significantly smaller (*p* < 0.001) than the 91.15 ± 0.47% obtained for MC. This may be due to the presence of lignin in the agroindustrial cellulose samples obtained in this study. As pointed out by Shebani et al. (2008), the ash and lignin contents are related because, as this component degrades at temperatures higher than hemicellulose and cellulose, between 250 and 500 °C, its combustion does not occur until 600 °C, leaving a higher residue content after combustion, at this temperature.

### Morphological analysis of cellulose

**Fig. 6:**
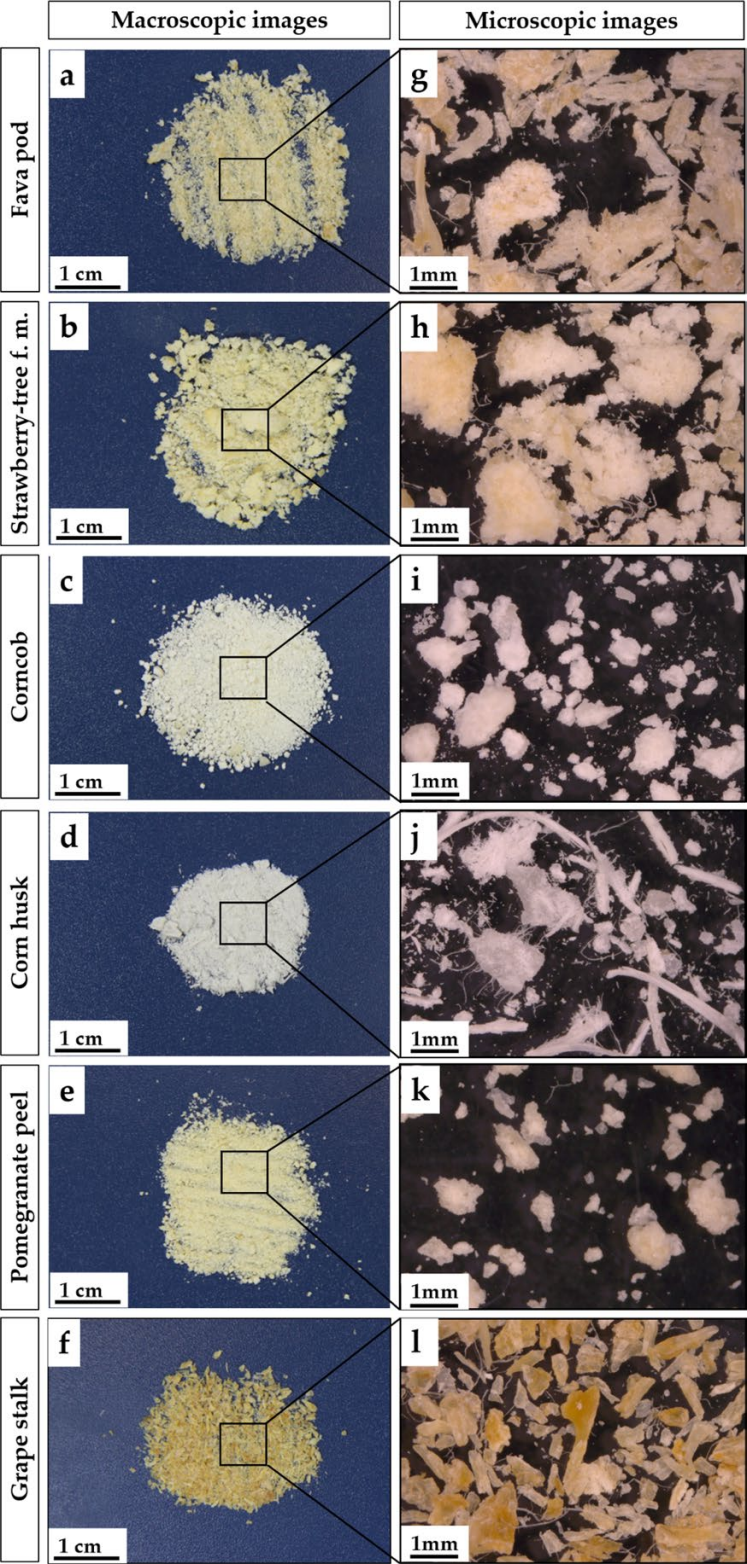
Macroscopic (**a**–**f**) and microscopic (**g**–**l**) images (20× amplification) of the extracted cellulose samples

The macroscopic and microscopic images of the cellulose samples obtained in this study indicate noticeable structural differences among the cellulose samples obtained from the different agroindustrial residues. The most evident differences were the color and brittleness (Fig. 6a–f) which are two features mainly associated with the residual presence of lignin (Rojas et al. 2015) that was possibly not properly removed during the bleaching step. The obtained celluloses can be classified from yellow to white, according to their origin, by the following order: grape stalk, fava pod, strawberry-tree fruit marc, pomegranate peel, corncob and corn husk. These observations suggest that the bleaching process was more efficient in the case of corncob and corn husk. A previous work that investigated the fractionation of cellulose, hemicelluloses and lignin from grape stalk, already mentioned a general difficulty in obtaining well delignified cellulose fractions from this residue, highly influenced by the cultivar (Spigno et al. 2008). It is also important to refer that the organization of the lignin molecules within the raw material cell wall structure, such as the associative interactions with other cell wall components like cellulose, is determinant to make the extraction process more or less efficient. According to this, if color and cellulose purity are important features, the bleaching conditions must be optimized and adapted to the nature of the agroindustrial residue.

Considering the visual appearance of the samples, it is evident the presence of cellulose aggregates in all cases. The production of individualized particles of cellulose is challenging because of the strong agglomeration tendency which occurs upon drying cellulose suspensions (Rojas et al. 2015). To decrease aggregation, lyophilization is recommended over oven-drying. The raw materials with a more homogeneous morphological distribution of cellulose aggregates were grape stalk (Fig. 6f), pomegranate peel (Fig. 6e) and corncob (Fig. 6c). Regarding corn husk cellulose (Fig. 6j), two very distinctive aggregate shapes were found: round-like aggregates, very similar to the ones obtained for corncob and pomegranate peel celluloses, and long fiber shape aggregates. This last type of aggregates was also observed in the case of fava pod cellulose (Fig. 6g). Noticeably, these two cellulose samples with long fiber aggregates were also the ones that presented higher degradation temperatures, reaching 340 °C (Table 2). Grape stalk cellulose (Fig. 6l) presented the most crystal-like aggregates which, as mentioned before, may be associated with the presence of residual lignin.

**Fig. 7:**
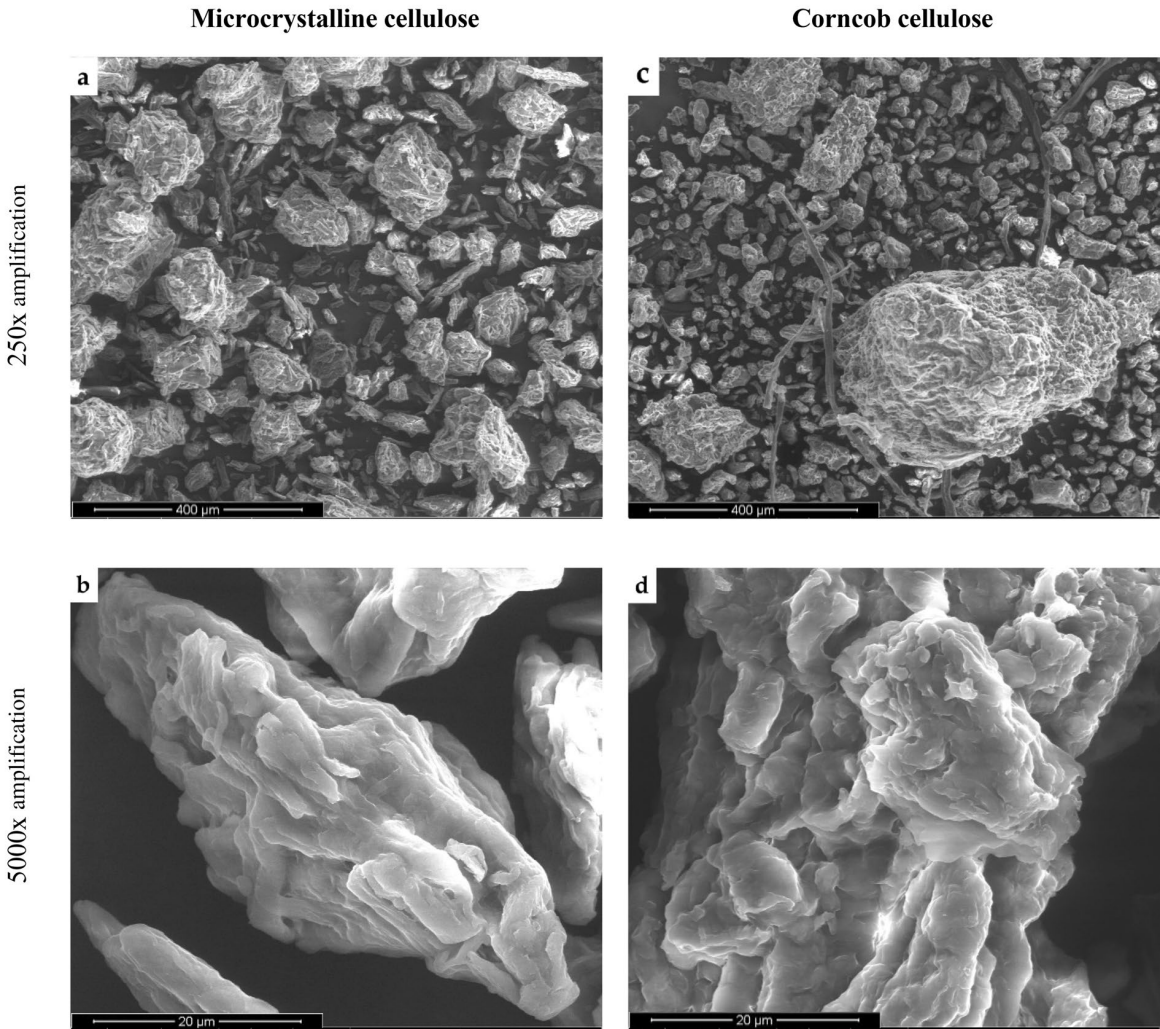
SEM images of MC and corncob cellulose for different amplifications

Given that the extraction of cellulose from corncob reached the highest extraction yield (26.1 ± 1.2%, Fig. 3) and that it was the only sample where the presence of lignin and hemicellulose was not confirmed in the FTIR analysis, its structure was further analyzed by SEM and compared to MC. Figure 7 shows the SEM images under two different magnifications and reveals differences in the morphology of the two samples. Considering the lowest magnification (250×), MC presented a more homogeneous particle size distribution (Fig. 7a), while corncob cellulose showed a more heterogeneous morphology with smaller and bigger particle aggregates/clusters and some macrofibrils (Fig. 7c). The micrographs obtained with the highest magnification (5000×) (Fig. 7b, d) showed that MC has a compact surface morphology with a smoother surface. The irregularities observed at the surface of the corncob cellulose may be a result of the severity of the treatment that extensively degraded the amorphous regions of cellulose, considering that it was extracted from an agroindustrial residue, which is considered a weaker lignocellulosic material comparatively to wood-derived lignocellulosic raw materials (García et al. 2014). However, and at this level of detail, it could be observed that in general terms both cellulose structures were similar, despite their different origins.

## Conclusions

In this study, agroindustrial residues of corn, grape, pomegranate, strawberry-tree fruit and fava were subjected to a multi-step extraction procedure in order to isolate cellulose with potential to be used in fiber-reinforced composite scaffolds for biomedical applications. The recovery of purified cellulose was successful, with yields varying from 4.8% for pomegranate peel to 26% for corncob. FTIR analysis confirmed that the multi-step extraction procedure was successful in isolating cellulose from the six agroindustrial residues, as the spectra presented the typical peaks of cellulose. The FTIR spectra also showed that trace amounts of lignin and hemicellulose may possibly be present in all the obtained cellulose samples, with the exception of the one derived from corncob. The thermogravimetric analysis confirmed the identical thermal behavior of the agroindustrial residues celluloses and the wood-derived MC used as a control, with degradation temperatures in the range 330–340 °C as expected for cellulose-rich materials. Macroscopic and microscopic analyses showed notorious physical differences among the studied celluloses, with SEM confirming high morphological similarities between corncob cellulose and MC.

In conclusion, this study suggests novel non-wood cellulosic biomass sources, thus contributing to the development of new trade opportunities for the involved processing industries, and following the current worldwide tendency of recycling agroindustrial residues.

## Data Availability

The datasets used and analyzed during the current study are available from the corresponding author on reasonable request.

## Abbreviations

ATR-FTIR: Attenuated total reflectance Fourier-transform infrared spectroscopy
DSC: Differential scanning calorimetry
MC: Microcrystalline cellulose
SEM: Scanning electron microscopy
TGA: Thermal gravimetric analysis
